# Assessing the Genetic and Environmental Factors on Egg Amino Acid Traits in Chickens: A Review

**DOI:** 10.3390/ani15111554

**Published:** 2025-05-26

**Authors:** Dipson Gyawali, Tatsuhiko Goto

**Affiliations:** 1 Department of Life and Food Sciences, Obihiro University of Agriculture and Veterinary Medicine, Obihiro 080-8555, Hokkaido, Japan; deepsangyawali11@gmail.com; 2 Research Center for Global Agromedicine, Obihiro University of Agriculture and Veterinary Medicine, Obihiro 080-8555, Hokkaido, Japan

**Keywords:** amino acid, chicken, egg, environment, food production, genetics, indigenous breed

## Abstract

Eggs are good sources of animal proteins. Methods for creating designer eggs are attractive to scientists and producers of the eggs, in order to fulfill customers’ demands. We review and summarize the current understanding of how genetic and environmental factors influence chicken egg components, specifically, amino acid contents of yolk and albumen. Genetically diverse breeds of chicken enable us to obtain a wide variety of egg amino acids. Moreover, the use of fermented feed and different rearing systems (barn and deep litter) has impacts on egg amino acids, which may be mediated through changes in the intestinal environment, including the microbiota. To overcome the future food crisis, the combinations of breeds, feeds, and rearing systems will be important for producing designer eggs. In the future, not only long-term selected layer strains will be required, but also many kinds of indigenous chicken breeds, which have already adapted to each local environment in the world, should be investigated under the continental levels of climate environment using different fermented feed materials. To better understand the interplay between genetic and environmental factors, we will offer valuable insights for both egg producers and consumers, potentially guiding future efforts to optimize egg amino acid contents in chickens.

## Simple Summary

This review discusses insights into the genetic and environmental influences on egg amino acid traits in chickens. It highlights that the differences in breeds, feeds, and rearing systems have the potential to alter egg components. The paper also addresses future prospects including genetic mapping, gut microbiota, and genetic–environmental interaction in the future food production.

## 1. Introduction

Eggs have good nutritional value, since they contain essential nutrients including protein, carbohydrates, fat, vitamins, and minerals [1]. In addition, egg components show a variety of biological functions, including antioxidant characteristics, antibacterial activity, immunomodulatory, anticancer, and antihypertensive activity [1,2]. The protein quality of eggs is known to be high due to their complete amino acid profile, which leads to valuable nutrition for human health [3]. Eggs are good sources of animal proteins that support the synthesis and repair of skeletal muscles in humans [4]. Designer eggs in designer foods are defined as altered concentrations in some components from regular eggs [5]. Scientists are trying to find methods for creating designer eggs to fulfill customers’ demands [6,7].

Various egg traits, including egg weight, eggshell thickness, yolk size, and albumen weight, are closely involved with the quality and quantity of the eggs, hence, factors affecting egg traits are of great interest in the poultry industry [8,9]. Egg quality traits are generally classified into two types, external and internal, which consist of egg weight (EW), length of the long axis of the egg (LLE), length of the short axis of the egg (LSE), eggshell weight (SW), eggshell strength (SS), eggshell thickness (ST), eggshell color lightness (SCL), eggshell color redness (SCR), eggshell color yellowness (SCY), albumen weight (AW), and yolk weight (YW) [10,11,12]. Egg quality traits are influenced by both genetic and environmental factors [9,13,14]. Heritability estimates of external and internal egg quality traits range from 0.30 to 0.70 [15,16,17], which implies that around 50 ± 20% of the phenotypic variances are influenced by genetic and environmental factors. Therefore, both factors are important for controlling the external and internal egg quality traits [18].

In terms of egg contents, Goto et al. [19] have investigated how chicken egg components (metabolites) are changed by breed and feed using a metabolomics approach. For the genetic factor (breed), egg metabolomes have been compared among three diverse West African chicken breeds [20] and Chinese local breed and commercial breeds [21]. Regarding the environmental factor (feed), Ogura et al. [22], Giannenas et al. [23], and Li et al. [24] have analyzed egg metabolites. Studies using a non-targeted metabolomics approach [25] have paid attention to understanding what factors affect egg components for creating designer eggs [19]. In comparison with the non-targeted approach, fatty acids in yolk are of great concern when using a targeted approach in animal nutrition and management. Eicosapentaenoic acid (EPA) and docosahexaenoic acid (DHA) (unsaturated fatty acids) enhanced eggs can be produced by feeding microalgae and fish oil [7]. Essential fatty acids added to designer eggs are well-known to improve human health [6].

Food functions have three categories: nutrition (primary function), palatability (secondary function), and bioregulation (tertiary function) [26]. Amino acids consist of protein, which is one of the essential nutrients. Free amino acids contribute to the taste of foods [27,28], which is related to palatability. In 1 g of freeze-dried egg yolk sample, the free amino acids make up approximately 10 mg [29]. Therefore, factors for altering amino acid contents in egg yolk and albumen will lead to the egg production of taste-added eggs [2]. In fact, Goto et al. [30] have reported that genetic and environmental factors can alter not only amino acid contents but also sensor values of bitterness in egg yolk and albumen, indicating that the combination of breed and feed will be crucial to make amino acids-enriched and taste-added designer eggs.

Amino acids can be obtained through feed digestion and absorption, tissue decomposition, and internal synthesis [31,32]. The metabolism and transportation of amino acids are performed in many tissues, including the digestive tract, small and large intestines, blood vessels, liver, ovary, and oviduct [33,34,35]. The gut microbial metabolism of amino acids influences the amino acid metabolism in the host [36]. Yolk and albumen are produced in several tissues including the liver, ovary, and oviduct. Yolk components, which are mainly created in the liver, are accumulated in the follicle of the ovary, whereas albumen components are secreted from the oviduct magnum [37,38]. It is well known that many genes expressed in the ovary and magnum are involved in chicken egg formation, which is supported by combining genomics, transcriptome, and proteome data [39].

The purpose of this review article is to summarize the current understanding of how genetic and environmental factors influence chicken egg components, specifically focusing on the amino acid contents of yolk and albumen, which are mainly investigated by our group. We will highlight the significant role of genetic factors in modifying free amino acids in eggs and discuss how environmental interventions can alter amino acid traits. Additionally, we will evaluate the interplay between genetic and environmental factors in modifying egg amino acid traits. This review will offer valuable insights for egg producers and consumers, potentially guiding future efforts to optimize egg amino acid contents in chickens.

## 2. Amino Acid Metabolism in the Eggs

Amino acids known to be important not only for protein synthesis, but also for generating glucose, ATP, fatty acids, and metabolic precursors for biomolecules, including heme, nucleotides, catecholamines, and neurotransmitters [35]. The animal body can obtain amino acids through feed digestion and absorption, tissue decomposition, and internal synthesis [31]. Amino acid metabolism can be promoted by digestion using amino acid degrading enzymes and intestinal microbiota [33,40], absorption mediated by amino acid transporters [33,41,42], and nutrient transport in the bloodstream [34,35]. Although the absorption of amino acids is largely completed in the small intestine, the uptake of amino acids derived from bacterial metabolism and endogenous sources is mediated by the large intestine [33]. The cellular uptake of amino acids is involved in amino acid transporters, which serve as the entry and exit channels of amino acids and act as probes for sensing amino acid concentrations [31,43,44].

Amino acid degradation produces ammonium, which is removed by the synthesis of nitrogen-containing compounds (nucleotides), or excreted in the form of urea via the urea cycle [35]. The carbon skeletons of amino acids can be converted into TCA cycle intermediates, which are used in oxidative phosphorylation, fatty acid synthesis, and gluconeogenesis [35]. Amino acid catabolism occurs mainly in the liver and is involved in the intestinal microbiota [34,40]. The gut microbial metabolism of amino acids plays an important role in host-microbe interactions [36]. The consumption of a high-protein diet tends to accumulate protein-fermenting bacteria and reduce the proportion of saccharolytic bacteria [40]. Thus, the feeding environment will influence amino acid metabolism through digestion, absorption, and nutrient transport.

Egg components (yolk, albumen, eggshell membrane, and eggshell) are produced in several tissues, including the liver, ovary, and oviduct. The yolk precursors are mainly synthesized by the liver, secreted into the blood, and then transported to the left ovary [37,39]. After forming the primary follicle by rapid growth of a single oocyte in the ovary, ovulation occurs and the yolk travels down the oviduct [38,45]. Magnum, isthmus, and uterus (shell gland) in the oviduct are known as the parts of the oviduct with secretory glands for albumen, eggshell membrane, and eggshell, respectively [38,39,46]. Yin et al. [39] have revealed hundreds of genes that are differentially expressed in the ovary, magnum, isthmus, and uterus using integrated ovarian and oviduct transcriptomes by mRNA sequencing. This implies many genes are candidates to regulate the contents of amino acids in yolk and albumen.

## 3. Analytic Methods

The amino acids can be analyzed using a variety of technologies [47], including high-performance liquid chromatography (HPLC) [48], ultra-high-performance liquid chromatography (UHPLC) [49], amino acid analyzer [50], ultra-high-performance liquid chromatography–mass spectrometry (UHPLC-MS) [51], capillary electrophoresis–mass spectrometry (CE-MS) [22], gas chromatography–mass spectrometry (GC-MS) [19], and liquid chromatography–mass spectrometry (LC-MS) [52]. Sample pre-preparation is required to avoid large molecules from samples before using the equipment. Raw samples and freeze-dry samples of the yolk and albumen are conducted deproteinization and filtered by a membrane filter unit with less than 0.45 μm pore size [2].

Non-target metabolomics can detect several amino acids, but peak sizes of the other amino acids tend not to be high enough from the baseline noise. The target metabolomics approach can analyze the contents of amino acids with high detection sensitivity. In addition, pre-column and in-column derivatization makes the peaks highly sensitive [29,30]. Egg amino acids are chemically modified through pre-column derivatization using phenylisothiocyanate (PITC) and ortho-phthalaldehyde (OPA) and then measured via ultra-violet (UV) and fluorescence detectors, respectively [2,49]. Ninhydrin colorimetric analysis is used by an automated amino acid analyzer [50]. Trimethylsilyl (TMS) derivatives are used in metabolomics [19]. Moreover, stable isotope dilution analysis combined with mass spectrometry, a highly effective method, is widely used in quantitative analysis for the determination of food components [53]. Liquid chromatography–tandem mass spectrometry (LC-MS/MS) with stable isotope dilution analysis of methylhistidine in chicken plasma has achieved high inter-day and intraday repeatabilities [52].

Amino acids can be detected in egg yolk and albumen in chickens. They include aspartic acid (Asp), glutamic acid (Glu), asparagine (Asn), serine (Ser), glutamine (Gln), glycine (Gly), histidine (His), arginine (Arg), threonine (Thr), alanine (Ala), proline (Pro), gamma-aminobutyric acid (GABA), tyrosine (Tyr), valine (Val), methionine (Met), isoleucine (Ile), leucine (Leu), phenylalanine (Phe), lysine (Lys), cysteine (Cys), and tryptophan (Trp), through pre-column derivatization using PITC and OPA via HPLC and UHPLC, respectively [2,49,54,55]. Goto et al. [30] have confirmed high detection sensitivity in these amino acids, in comparison with our non-target metabolomics approach [19].

Analytical methods for the determination of amino acids in biological samples have been summarized through past, present, and future [56]. Analytic methods, which were not included in the present article, especially on-chip analysis using microfluidic devices allow us to achieve high integration, automatic operation, and lower sample and reagent consumption [57]. Comparisons of analytic methods for amino acids have been summarized in several review papers [57,58,59,60], which include the sample preparation, analysis time, derivatization, separation method, and sensitivity. The advantages and disadvantages of several analytic methods are found in the amino acids [58] and branched-chain amino acids (BCAAs) [60]. Therefore, we can select the analytic methods depending on the cost of equipment and reagents, sensitivity, reproducibility, and separation speed.

## 4. Genetic Factors

### 4.1. Breed/Strain Comparisons

**Figure 1:**
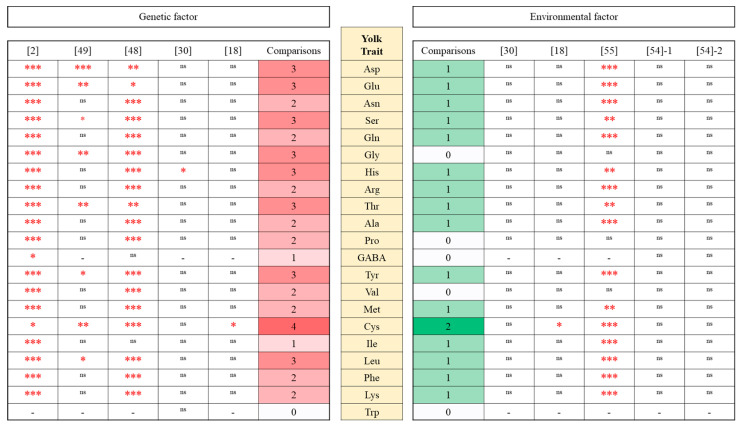
Summary of genetic and environmental influences in egg yolk amino acids. Goto et al. [2] used five breeds and two F_1_ hybrids of chickens under a cage environment. Nishimura et al. [49] used five breeds of chickens in floor rearing environment. Goto et al. [48] used three breeds of chickens in a cage environment. Goto et al. [30] and Mori et al. [18] used two breeds of chickens and two kinds of feed under a cage environment. Kawamura et al. [55] used cage and floor environments. Kawamura et al. [54]-1 and Kawamura et al. [54]-2 used cage and litter environments in the first and second stages, respectively. Comparisons: number of comparisons with significant effect. *p* value of ANOVA for genetic or environmental effect (*** *p* < 0.001, ** *p* < 0.01, * *p* < 0.05, and ^ns^ *p* > 0.05) in each comparison. - Not available.

**Figure 2:**
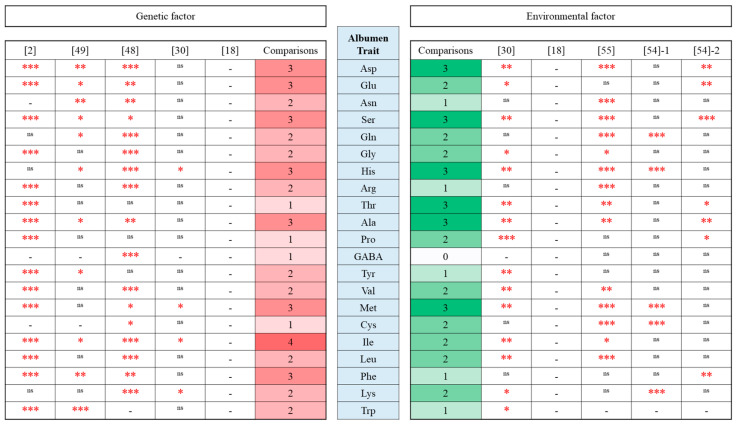
Summary of genetic and environmental influences in egg albumen amino acids. Goto et al. [2] used five breeds and two F_1_ hybrids of chickens under a cage environment. Nishimura et al. [49] used five breeds of chickens in floor rearing environment. Goto et al. [48] used three breeds of chickens in a cage environment. Goto et al. [30] and Mori et al. [18] used two breeds of chickens and two kinds of feed under a cage environment. Kawamura et al. [55] used cage and floor environments. Kawamura et al. [54]-1 and Kawamura et al. [54]-2 used cage and litter environments in the first and second stages, respectively. Comparisons: number of comparisons with significant effect. *p* value of ANOVA for genetic or environmental effect (*** *p* < 0.001, ** *p* < 0.01, * *p* < 0.05, and ^ns^ *p* > 0.05) in each comparison. - Not available.

Genetic differences in egg quality traits are obvious between breeds/strains within the species [61,62]. Many studies have revealed significant breed effects on various egg characteristics [12]. For egg amino acid traits, our team has reported significant effects on breed/strain in the yolk (Figure 1) and albumen (Figure 2). Goto et al. [30] and Mori et al. [18] have analyzed breed difference in yolk and albumen amino acids using eggs from Rhode Island Red and Australorp breeds (Western breeds) under a cage rearing system. Significant breed effects were observed in two yolk amino acids (His and Cys) and four albumen amino acids (His, Met, Ile, and Lys). Goto et al. [2] used five breeds (Australorp, Rhode Island Red; RIR, Nagoya; NGY, Shamo; SHA, and Ukokkei; Western breeds and Japanese indigenous breeds) and two F_1_ hybrids (NGYxRIR and SHAxRIR) to know effects on the genetic difference in yolk and albumen amino acids under a cage rearing system. Significant effects on genetic difference were found in 20 yolk amino acids (Asp, Glu, Asn, Ser, Gln, Gly, His, Arg, Thr, Ala, Pro, GABA, Tyr, Val, Met, Cys, Ile, Leu, Phe, and Lys) and 15 albumen amino acids (Asp, Glu, Ser, Gly, Arg, Thr, Ala, Pro, Tyr, Val, Met, Ile, Leu, Phe, and Trp). Nishimura et al. [49] tested egg amino acids using Ukokkei and Nagoya (Japanese indigenous breeds) and Araucana cross, Kurohisui, and Boris Brown (three hybrids) under a floor-rearing system. Significant effects of genetic difference were seen in 8 yolk amino acids (Asp, Glu, Ser, Gly, Thr, Tyr, Cys, and Leu) and 11 albumen amino acids (Asp, Glu, Asn, Ser, Gln, His, Ala, Tyr, Ile, Phe, and Trp). Goto et al. [48] used Nagoya and Yakido (Japanese indigenous breed) and Boris Brown (hybrid layer) for comparison of egg amino acids under a cage-rearing system. Significant effects on genetic difference were detected in 18 yolk amino acids (Asp, Glu, Asn, Ser, Gln, Gly, His, Arg, Thr, Ala, Pro, Tyr, Val, Met, Cys, Leu, Phe, and Lys) and 17 albumen amino acids (Asp, Glu, Asn, Ser, Gln, Gly, His, Arg, Ala, GABA, Val, Met, Cys, Ile, Leu, Phe, and Lys).

### 4.2. Yolk Amino Acids

In Figure 1, we can observe which yolk amino acids tend to change by genetic difference using five times comparisons. The 20 kinds of amino acids, except for Trp, can be altered significantly, even in different environmental conditions including feed material and rearing systems. Of them, nine yolk amino acids (Asp, Glu, Ser, Gly, His, Thr, Tyr, Cys, and Leu) are frequently changeable by genetic factors. Although the comparison within Western breeds tends to show almost no genetic effect [18,30], the comparisons between Japanese indigenous breeds and Western breeds tend to have huge power to change many kinds of yolk amino acids [2,49]. This will indicate that the degree of genetic divergence positively correlates with the number of yolk amino acids significantly altered. Moreover, this implies that many genetic variants regulate amino acid contents in the yolk.

Li et al. [63] have tested yolk amino acid traits using two divergent broiler lines (lean and fat lines), which have a selective history of abdominal fat percentage since 1996. An amino acid analyzer detected 17 amino acids including Gly, Ala, Asp, Glu, Leu, Lys, and Pro in the yolk. No significant difference between the lines was found in the yolk amino acids.

### 4.3. Albumen Amino Acids

Figure 2 summarizes which albumen amino acids tend to change through genetic differences with four times comparisons. All 21 kinds of amino acids can be changed significantly, even in the different conditions of feeding and rearing. The eight yolk amino acids (Asp, Glu, Ser, His, Ala, Met, Ile, and Phe) are frequently changeable through genetic factors. As well as yolk amino acids, Japanese indigenous breeds and Western breeds and their hybrids indicate wider phenotypic diversity in many kinds of albumen amino acids [2,49]. These results imply that albumen amino acids will be regulated by many genetic variants with small effects of a quantitative nature [9].

In the study of Li et al. [63], albumen amino acid traits were compared using two divergent broiler lines (lean and fat lines). The 17 amino acids including Gly, Ala, Asp, Glu, and Leu were detected in the albumen. Significant differences between the lines were found in Arg, Asp, Gly, His, Leu, Met, and Thr in the albumen. The amino acids of the fat line were higher than those of the lean line.

### 4.4. Nutrient Transport

Genetic differences among breeds/strains would significantly influence amino acids in eggs, primarily through variations in feed digestion and absorption, nutrient transport, and regulatory pathways affecting ovarian and oviduct function. The impact of breed on gene expression of amino acid transporters was clarified by Zeng et al. [64] using intestinal samples collected on embryonic days 9, 12, 14, 17, and 19 and the day of hatch. The study revealed significant differences in the expression of amino acid transporters between the Wenshi Yellow-Feathered chick (WYFC) and the White Recessive Plymouth Rock chick (WRRC). Specifically, the mRNA levels of the five amino acid transporters were significantly higher in the slow-growing WYFC than in the WRRC. In addition, Li et al. [65] have studied the embryonic development and gene expression for amino acid transporters in two breeds with different growth rates, White Plymouth Rock (WPR) and WENS Yellow Feather Chickens (WYFC). Breed-specific differences were observed for solute carrier (SLC) superfamily of transporters (SLC7A9, SLC1A1, and SLC15A1) gene expression in small intestines at embryonic development.

The gene expression of nutrient transporters has been investigated in different intestinal segments in uninfected Ross Heritage broilers and Sexsal layers at 27 days of age [66]. In this study, the expression levels of cationic amino acid transporter-2 in the jejunum and ileum and L-amino acid transporter-1 in the ileum were significantly higher in the layer than in the broiler. In addition, Miska and Fetterer [67] investigated nutrient processing and uptake between modern fast-growing broiler line (Ross) and slow-growing broiler line (ACRBC), which have not been selected for rapid growth since 1957, using gene expression levels in the duodenum, jejunum, and ileum at 1, 3, 5, 10, and 14 days of age. As a result, the genes of brush border amino acid transporters, which are responsible for bringing in amino acids into the enterocyte from the gut lumen, were significantly expressed in ACRBC birds relative to Ross birds, especially at earlier time points. They mentioned the possibility that the slow-growing ACRBC has much smaller amounts of nutrients in the gut than the fast-growing Ross, and therefore more amino acid transporters are necessary to bring these nutrients into the cells.

## 5. Environmental Factors

### 5.1. Yolk and Albumen Amino Acids

Environmental differences including feed and rearing systems are also major issues in egg quality traits [8]. Many studies have revealed significant feed effects on various egg characteristics including yolk fatty acids [7]. For egg amino acid traits, our team has reported significant effects on the feed and rearing system in yolk and albumen (Figure 1 and Figure 2). Goto et al. [30] and Mori et al. [18] have analyzed feed effects in yolk and albumen amino acids using fermented feed and mixed feed under a cage-rearing system. Significant feed effects on a yolk amino acid (Cys) and 15 albumen amino acids (Asp, Glu, Ser, Gly, His, Thr, Ala, Pro, Tyr, Val, Met, Ile, Leu, Lys, and Trp) were observed. Kawamura et al. [55] investigated combined effects on the rearing system (cage and barn) and feed crude protein (CP 15.5% and CP 17.0%) in yolk and albumen amino acids. Significant combined effects on rearing and feeding CP were detected in 16 yolk amino acids (Asp, Glu, Asn, Ser, Gln, His, Arg, Thr, Ala, Tyr, Met, Cys, Ile, Leu, Phe, and Lys) and 14 albumen amino acids (Asp, Asn, Ser, Gln, Gly, His, Arg, Thr, Ala, Val, Met, Cys, Ile, and Leu). Since additional investigation clearly indicated there are no effects on feed CP (CP 15.5% and CP 17.0%) in the cage system, the major contribution of the combined effects will be derived from different rearing systems (cage and barn). Although feed crude protein would be thought to be the source of egg amino acids, the study indicated that the slight CP percentage difference (1.5%) seems to have no power to change egg amino acid contents. The evidence implies that there is a relatively complex system for digestion, absorption, and metabolism of amino acids from feed to egg. Kawamura et al. [54] have studied using Tosa-jidori, a Japanese indigenous breed, to test how the rearing system (cage and deep litter) affects egg amino acids. Although there was no significant effect on yolk amino acids in two comparisons, significant rearing effects were found in 12 albumen amino acids (Asp, Glu, Ser, Gln, His, Thr, Ala, Pro, Met, Cys, Phe, and Lys).

We can see which yolk amino acids tend to change by environmental difference using five times comparisons (Figure 1). The 16 yolk amino acids, except for Gly, Pro, GABA, and Val, can be significantly altered by feed and rearing. However, there were no amino acids that frequently changed in the yolk. On the other hand, the 20 kinds of albumen amino acids except for GABA can be altered significantly, even in the different breed/strain conditions (Figure 2). Of them, six albumen amino acids (Asp, Ser, His, Thr, Ala, and Met) are frequently changeable by environmental factors. The results of genetic and environmental effects on albumen amino acids clearly tell us the possibilities that selecting options of feed and rearing system enable us to alter albumen amino acids more easily than yolk amino acids (Figure 1 and Figure 2).

Li et al. [63] compared the yolk and albumen amino acids using two maternal dietary intake groups (normal and low maternal dietary intake), 100 and 75% of the dietary intake recommended by the Chinese Ministry of Agriculture. Even though the feed allocation (g/bird/day) was 25% different, no difference was found in the albumen amino acids. Although the low maternal dietary intake group implies a low intake of sources of amino acids, the low maternal dietary intake significantly increased the cystine in the yolk [63]. The lines of evidence will indicate that it is slightly difficult to alter egg amino acids by changing feed quality (CP level) and quantity (daily allocation) in basal diets.

### 5.2. Microbiota Influence in Amino Acid Metabolism

The gut microbiota is considered one of the key elements to regulate host health [68]. Gut microbiota and microbial pathways influence the metabolism of dietary carbohydrates (to short chain fatty acids and gases), proteins, plant polyphenols, bile acids, and vitamins [69]. Environmental interventions, such as altered diet menus and exercise habits, are known modulators of gut microbiota [70,71]. Microbial communities at higher taxonomic levels are very similar between mammals and avian; two phyla, *Firmicutes* and *Bacteroidetes*, are dominant out of 75 known microbial phyla [72]. In chickens, the potentially different amount of exercise using cage and litter rearing systems altered gut microbiota [73]. In addition, the feed additive affects microbiota in chickens [74,75]. Dietary rosemary ultrafine powder significantly impacted both the alpha- and beta-diversity of the caecum microbiota in Rhode Island Red [76], whereas the supplementation of sodium butyrate regulated NH_3_-producing bacteria to reduce NH_3_ production in the cecum of laying hens [77]. The litter, the bedding materials used in the floor housing system, is usually mixed with chickens’ excreta with a complex microbial community and thus has a potential impact on chicken gut microbiome [74]. The presence of excreta (directly in contact with feces) in floor litter housing system altered gut microbiota in comparison with the plastic net housing system [78].

Fermented foods, made through desired microbial growth and enzymatic conversions of food components, could benefit health through the nutritive alteration of the ingredients, modulation of the immune system, and the presence of bioactive compounds that affect intestinal and systemic function [79]. Fermentation-associated microorganisms, including yeast, lactic acid bacteria, *Bacillus*, and molds, might alter the intestinal composition or function of the autochthonous microbiota in the gastrointestinal tract [80]. Kumar et al. [81] summarized that functional fermented foods have the role of probiotics, prebiotics, and synbiotics on animal health, which affect gut microbiota and overall gut environment such as the pH, temperature, and concentration. Microbiota influence not only energy-delivering macronutrients (e.g., fat, carbohydrate, protein) but also essential nonenergy-delivering micronutrients (e.g., vitamins, minerals, trace elements), which indicate the importance of host–microbe–metabolic axis and their impact on health for attenuating hidden hunger [82]. In chickens, fermented feeds create a symbiotic relationship with microbiome that enhances amino acids availability through three primary mechanisms, microbial proteolysis, enhanced nutrient absorption, and bacterial synthesis of bioactive compounds [83,84,85]. Chalvon-Demersay et al. [86] summarized main metabolites including short chain fatty acids and polyamines, produced by amino acid metabolism via the gut microbiota and associated effect on gut health. Since the microbiota affect amino acid metabolism [33,40], the altered egg amino acids using different feed and rearing systems [18,19,54,55] might be involved in the gut microbiome change.

## 6. Conclusions and Future Prospects 

This review article mainly focused on how chicken egg amino acids can be modified by genetic and environmental factors using several comparisons. Since the egg amino acids that show quantitative phenotypic variances are influenced by both genetic and environmental factors [9], there will not be a single locus with a large effect such as the Mendelian locus. Until now, there has been almost no evidence about heritability estimates and genetic loci for egg yolk and albumen amino acid traits in chickens. Therefore, our research team is creating several quantitative trait loci (QTL) mapping populations including multi-parent population, e.g., multi-parent advanced generation intercross (MAGIC) population [87,88], to discover many QTLs affecting yolk and albumen amino acid traits in chickens [9]. In addition, we are collecting genotypic and phenotypic data from a single-breed derived breeding population with pedigree control to estimate heritability and to find QTLs with genome-wide association study (GWAS). In the near future, several QTLs for yolk and albumen amino acid traits will be reported. Identifying QTLs for amino acid traits enables breeders to pinpoint specific genomic regions associated with desirable traits, such as enhanced amino acids. This information allows us to create new breeds/strains more efficiently by targeting individuals carrying favorable alleles in multiple loci [9], even before phenotypic traits are fully expressed. Because of the complex nature underlying genetic architecture, genetic mapping studies should be ideally conducted with not only industrial chicken populations (layer and broiler) but also genetically divergent indigenous chicken breeds, leading to a better understanding [9]. Accumulating evidence of the relationship between genotype and phenotype in egg amino acids will be crucial to reveal the genetic basis and future improvement of egg components by breeding scheme.

**Figure 3:**
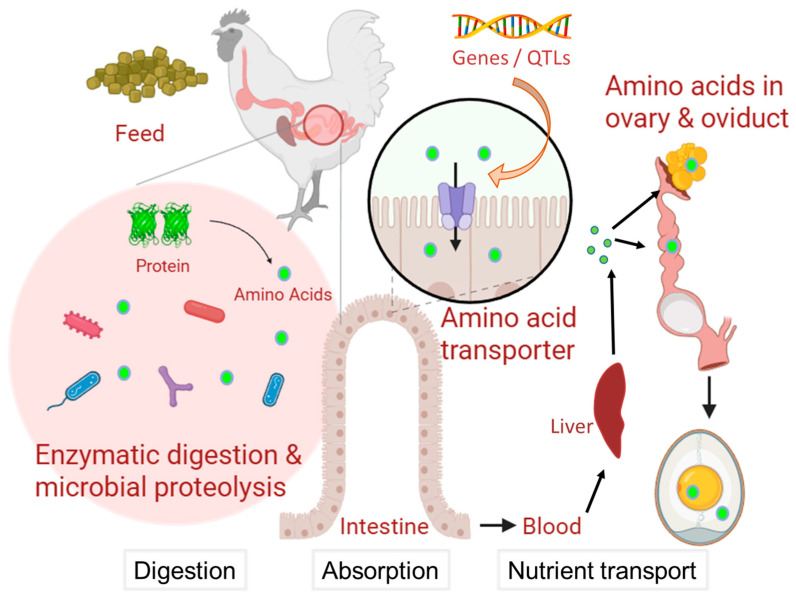
Potential mechanism of amino acid metabolism from feeds to eggs. After chickens eat feed materials, enzymatic digestion and intestinal microbial proteolysis occur in the gastrointestinal tract, from proteins to amino acids. The cellular uptake of amino acids is involved in amino acid transporters in the intestine. Amino acids are transported via the bloodstream to the liver, ovary, and oviduct. Yolk precursors are mainly created in the liver, and yolk components are accumulated in the ovarian follicles. At the oviduct magnum, albumen components are secreted. Thus, eggs contain various levels of free amino acids in the yolk and albumen. The host’s genes and/or QTLs broadly influence amino acid metabolism throughout the many processes of digestion, absorption, and nutrient transport. For example, the potential variants around amino acid transporter genes will alter the transportation efficiency of amino acids, leading to altered concentrations of egg amino acids. The genetic (breeds/strains) by environmental (fermented feed derived effects of probiotics and prebiotics) interaction with microbial influence will enhance nutritional levels of the eggs. The figure was created using BioRender.com (https://www.biorender.com/) (accessed on 1 May 2025).

To manage the contents of egg amino acids, it is also important to use environmental factors. Historically, much research has been performed to find what conditions of feeding materials and additives affect egg components, especially fatty acids [7]. In our previous studies [18,19], we focused on the use of lactic acid bacteria and cellulolytic enzyme digested-fermented feed, which include wheat, pumpkin, yam, soybean, potato, rice bran, fish meal, beet lees, scallop shell, and other materials. Since significant effects of feed were found in 12 albumen metabolites and 3 yolk metabolites [19], the fermented feeds, which probably contain a richer microbiome than the conventional mixed feed, have a larger impact on albumen amino acids. These results imply that the gut microbial environment, which will be potentially changed by rich microbiome feed, leads to the production of altered egg amino acids. In addition, Kawamura et al. [55] revealed rearing system (cage vs. barn) has significant effects on altering yolk and albumen amino acids in layers, whereas Kawamura et al. [54] indicated that the rearing system (cage vs. deep litter) changed yolk and albumen amino acids in Japanese indigenous breeds. These results also imply that the gut microbial environment will be important in altering egg amino acids. In fact, there are several lines of evidence that gut microbiota is changed in different rearing systems including cage, dustbathing environment, plastic net, and litter [73,78,89,90,91]. The floor litter housing system had higher alpha diversity in the cecal and duodenal microbiota than the plastic net housing system in Shendan chickens [78], whereas the alpha diversity of ileum microbiota of the litter floor broilers was higher than the cage group [73]. These will indicate that different housing systems change the microbiota, which affects digestibility, nutrient absorption, metabolism, and egg formation [54] (Figure 3). Since environmental factors, especially the microbiome, probably have a large potential to modify egg components, not only egg components (final livestock product) but also intestinal flora and metabolism should be investigated [54].

The HungerMapLIVE (https://hungermap.wfp.org/) (accessed on 31 March 2025) indicated that there are 22 countries with very high levels of hunger, especially in Asian and African environments. To overcome the food crisis and to enhance the quality of life in humans [2], further production of chicken eggs with higher nutrients will be required. Although it is essential to use long-term selected egg layer strains for increasing the quantity of eggs, many kinds of indigenous chicken breeds, which have already adapted to each local environment in the world, should be explored for increasing the quality of eggs under the several conditions of feed and rearing system. The genetic and environmental interaction with microbial influence will play an important role in enhancing nutritional levels in the future (Figure 3). If we find a better combination between the local indigenous breeds (genetic factor) and local feed and rearing systems (environmental factor) to obtain higher nutritional eggs, the egg production system will adapt to each food production system at the continental level.

## Data Availability

Data are available upon request to the authors.
